# For the Good of the Globe: Moral Reasons for States to Mitigate Global Catastrophic Biological Risks

**DOI:** 10.1007/s11673-024-10337-z

**Published:** 2024-02-08

**Authors:** Tess F. Johnson

**Affiliations:** Ethox Centre, Nuffield Department of Population Health, University of Oxford, Big Data Institute, Old Rd, Oxford, OX3 7LF UK

**Keywords:** Global catastrophic biological risks, Global public goods, International justice, Cosmopolitanism, Nationalism, Bioethics

## Abstract

Actions to prepare for and prevent pandemics are a common topic for bioethical analysis. However, little attention has been paid to global catastrophic biological risks more broadly, including pandemics with artificial origins, the creation of agents for biological warfare, and harmful outcomes of human genome editing. What’s more, international policy discussions often focus on economic arguments for state action, ignoring a key potential set of reasons for states to mitigate global catastrophic biological risks: moral reasons. In this paper, I frame the mitigation of such risks as a global public good, and I explore three possible categories of moral reasons that might motivate states to provide this global public good: nationalism, cosmopolitanism, and interstate obligations. Whilst there are strong objections to moral nationalism as a reason for states to act, moral cosmopolitanism may provide a broad reason which is further supplemented for individual states through the elaboration of interstate moral obligations. The obligations I consider are moral leadership, fairness, and reciprocity. Moral reasons for individual states action may more effectively or more appropriately motivate states to mitigate global catastrophic biological risks.

## Introduction

Harms during the COVID-19 pandemic resulted not only from the disease itself, but from poorly internationally coordinated responses. Mortality, economic losses, and ongoing mental and physical health burdens continue around the world. The pandemic highlighted human vulnerability to biological threats, and policymakers’ often-ineffectual attempts to take collective action against them. In 2020–2021, worldwide excess mortality associated with COVID-19 was around fifteen million lives (World Health Organization 2022). Yet, even COVID-19 has not had a very significant impact on the world, when compared to the biological catastrophes that might be still to come. “Global catastrophic biological risks” (GCBRs) can be defined as events leading to “sudden, extraordinary, widespread disaster beyond the collective capability of national and international governments and the private sector to control” (Schoch-Spana, et al. 2017, 323). Examples may include naturally occurring pandemics, pandemics resulting from artificial/engineered pathogens, use of bioweapons programmes, the development of extreme drug resistance across multiple pathogens, or bio-hacking and harmful outcomes of human genome editing (Nouri and Chyba 2011). Their possible effects (whether the actions were deliberate or accidental) range from societal collapse to the institution of totalitarian regimes, to extreme morbidity and mortality across the human population. Indeed, they might occur on a scale that could cause human extinction and would then be re-termed existential risks. Whilst the risk of extinction from natural causes remains relatively constant (and we are not extinct yet, despite having spent a while now on this planet), anthropogenic existential and catastrophic risks are increasing. Despite this, bioethical work to date mostly focuses on natural pandemics and biological risks (Dawson 2007; Emanuel, et al. 2020; Giubilini 2019), with some exceptions (Adalja, et al. 2019; Chyba and Greninger 2004). Building on emerging public goods accounts in bioethics, I propose applying this framing to GCBRs to illuminate the issue of GCBR mitigation in a way that may help the future development and international coordination of interventions.

Experts on GCBRs have estimated a chance of up to 1 in 1000 that humanity will become extinct from an artificial pandemic within the next century (Lewis 2020; Millett and Snyder-Beattie 2017). This is an example of only one potential GCBR, and we might therefore expect the risk of extinction from GCBRs as a whole to be greater. The data is based on extrapolations from historical data on experiments with creating or modifying pathogens. The situation for this GCBR and others that involve the use of biotechnologies, including biohacking and misuses of human genome editing may deteriorate in the future, as two changes occur (Lewis 2020). The first change is increasing technological availability: the tools needed to edit genomes, whether pathogen or human, are becoming more available and affordable, with benchtop DNA synthesizers available to buy online, and mail-order DNA sequences available for USD 100-300 for a small gene, even back in 2017. The second change is knowledge availability: knowledge concerning how to simultaneously increase the lethality, transmissibility, incubation time and drug-resistance of pathogens whilst reducing their detectability is set to improve as synthetic biology research and, in particular, gain-of-function research continues and is performed and published in freely or pay-for-access academic journals. (For instance, in 2012, the U.S. National Science Advisory Board for Biosecurity allowed the publication of studies that described the modification of H5N1 viruses to allow airborne transmission between ferrets (Burki 2018).) The same goes for knowledge surrounding the influence of particular human genes on our traits and how these might be used for personal gain (Ma, et al. 2017).

Existing measures to mitigate GCBRs seem inadequate (Chyba and Greninger 2004; Kilbourne 2011). The main international agreement that might mitigate these risks is the biological weapons convention (BWC). The BWC aims to ban the development, storage, or use of biological and chemical weapons. In theory, this should prevent states party to the convention from causing a number of different types of GCBRs, and should require them to ensure their citizens are prevented from doing so. The BWC was introduced in 1972 and there are currently 185 states party to the convention (United Nations Office for Disarmament Affairs 1972). However, current levels of financial and structural support leave the BWC with a staff of only four people, and insufficient resources to verify state compliance or penalize non-complying states (Chyba and Greninger 2004). Whilst there are a number of other groups whose work supports the BWC, such as the United Nations Office for Disarmament Affairs, these do not play a direct role in monitoring and enforcement of the BWC. And whilst there may be other work being done that is not made public, if this exists, it does not appear too successful: there have been multiple violations of the convention (discovered or suspected) since its introduction (Lentzos 2019). What’s more, domestic structures for enforcement within nations appear scarce—for instance, scientists judge there to be too little regulation of the publication and performance of gain-of-function research in the United States and Europe (Fears and ter Meulen 2015; Kozlov 2022).

It may seem odd that states are not highly motivated to mitigate these risks, even without an international agreement. Mitigation would benefit both states’ own citizens, and everyone else—in fact, that may be part of the problem. When the benefits of a state investing in protection against a global threat befall everyone, there may be very little prudential or economic reason for purely self-interested states (that is, states that care only about their own citizens’ interests) to contribute to protection, rather than relying on others’ contributions to protect their own citizens (Aschenbrenner 2020; Millett and Snyder-Beattie 2017). Economic solutions and effective coercive mechanisms at the international level—which, as already noted, may be difficult to maintain (Martin 1999)—are unlikely to be successful, then. That said, there may be some possible solutions to mitigating GCBRs as a collective action problem (Weimann, et al. 2019), based on not merely economic but moral reasons.

My aim in this paper is to search for moral reasons to ground state decisions to undertake GCBR mitigation efforts. This work contributes another, perhaps more appropriate or effective motivating reason for GCBR mitigation than economic or political reasons. That motivation might impact policymakers directly or via a public who might (in a democratic state) vote for a party more willing to mitigate GCBRs (and there is evidence that moral arguments can change prospective voting behaviours, and thus, policymaking (Burstein 2003)). I begin by presenting an argument for framing GCBR mitigation as the production of a global public good (GPG) (section 1), which has not yet been thoroughly explored as a framing approach. By treating efforts to mitigate GCBRs as contributions to a GPG, I highlight three possible categories of moral reasons for contribution (section 2). The categories of moral reasons are: 1) moral nationalism—i.e., that regardless of whether contributing to GCBR mitigation constitutes production of a GPG that benefits everyone, more to the point it protects the citizens of a state, 2) moral cosmopolitanism—i.e., that concerning the production of GPGs like GCBR mitigation, states have obligations toward people outside their own jurisdictions, and 3) interstate obligations—i.e., that there are moral reasons for specific individual states to produce the GPG of GCBR mitigation based on obligations they hold toward other states, such as, moral leadership, fairness, and reciprocity. I conclude that states have moral reason to mitigate GCBRs, based foremost on their specific interstate obligations and an overarching reason of moral cosmopolitanism.

## GCBR Mitigation as a Global Public Good

The basis of my argument going forward is that GCBR mitigation constitutes a “global public good” (GPG). This is important both for explaining the weaknesses in an economic argument for states to contribute to mitigating GCBRs, and for grounding the categories of moral reasons for contributing to GCBR mitigation that I explore in the next section. I defend my claim that GCBR mitigation constitutes a GPG here by reference both to the definition of GPGs and how GCBR mitigation fits this, and by reference to similar examples of GPGs in the literature. In what follows in this section, I follow the status quo in the literature of treating states as individual, rational, self-interested economic actors coordinating to solve a public goods problem.

The standard definition of GPGs in economics differentiates them from other goods as a special category that raises particular problems and motivates particular behavioural strategies by states. Any good constitutes a GPG insofar as it has four characteristics that jointly render it 1) public and 2) global. Consider publicness, first. The benefits of a good are public if they are non-excludable and non-rivalrous (Hardin 1968; Samuelson 1954). That is, people cannot be prevented from accessing the benefits, and there is no less of the benefit left for others as a result of their accessing it. Another feature sometimes attributed to public goods is “the problem of jointness”—that is, the necessity of a large number of contributors for the production of the good (Waldron 1987). This raises problems of coordination that may not be seen with smaller groups, where more accountability is possible for (non-)contribution to the production of the good.

Consider globalness next. A GPG’s globalness is characterized by its aggregator technology and its spillover range (Kindleberger 1986). Aggregator technology refers to the marginal benefit produced by an additional contribution toward producing the GPG, and can be characterized as summative, weighted-sum, weakest-link, best-shot, or threshold, among others (as explored further below). A GPG may have one or a number of aggregator technologies associated with it. The spillover range refers to how far the benefits extend, and for a GPG, must include multiple global regions. Controversially, some definitions hold that a GPG’s spillover range should be not only geographically extensive but temporally extensive, too, such that future generations experience the benefit of GPGs produced today (Kaul, Grunberg, and Stern 1999).

GPGs face production problems—that is, where we consider states as individual, rational, self-interested economic actors, they will not coordinate to produce GPGs, at least in many scenarios. This is because of the non-excludability of their benefits. Whilst sometimes it is in an individual’s interest for a good to be secured even if others benefit from it, such as in economies of scale cases (Heath 2006), this is often not the case where the costs of contribution are moderate to high. Particularly in the case of global public goods, the logic of an n-prisoner’s dilemma features in a multiple-contributor public goods scenario, such that it is almost always in an individual state’s interest not to contribute to a public good and rather to free-ride, regardless of whether others are contributing (Hardin 1971). An n-prisoner’s dilemma occurs where multiple actors contribute resources to benefit from a shared good. Because there are many actors, they can each get away with free-riding on others’ contributions—that is, relying on others for the production of the public good without contributing. In the case where there are other altruistic states contributing, a state is incentivized to *not* contribute to GPG production. In this case, an individual state can increase its net benefit by eliminating the cost of contribution. This threatens the continued production of the GPG if many states stop contributing on the assumption they can free-ride. Second, even in the absence of altruistic others, states will be incentivized to not contribute to GPG production. Where other states will not contribute up to a threshold level for the good to be produced (at least in the case of GPGs with threshold aggregator technologies), an individual state’s own contribution may be futile, and thus a waste of resources. It is only in cases where there is significant marginal benefit from every contribution or where just one more state contribution is required to reach a threshold for GPG production where it will ever be in a state’s economic interest to contribute. Because of the n-prisoner’s dilemma set-up of public goods provision, economic arguments for contributions are weak motivators.

In framing GCBR mitigation as a GPG but setting aside economic arguments for coordination to produce this GPG, I hold that we might better consider moral reasons states have to contribute to GPGs and solve collective action problems. In social contract theory, mechanisms have been explored to solve such collective action problems, including economies of scale, gains from trade, risk-pooling, self-binding, and information transmission (Heath 2006). The extent to which these mechanisms constitute moral reasons varies, and their applicability to particular cases has not been thoroughly explored. Yet, in the bioethics literature, there has been a promising strategy of framing issues as public goods production problems and considering such mechanisms alongside other moral reasons for cooperation, particularly in the cases of population health (Anomaly 2011), herd immunity more specifically (Dawson 2007), procreation (Anomaly 2014), and protection of the antimicrobial commons (Giubilini 2019). It may be a promising framing strategy for highlighting obligations to contribute.

But does GCBR mitigation actually constitute a GPG according to the standard definition? GCBR mitigation is public. It produces non-excludable benefits (in most cases). Some GCBR mitigation might provide more benefits for some states than others—consider how states that develop and produce an essential vaccine may gain more from selling that vaccine (product or intellectual property) than the states that must buy it. However, in general, the benefit of not experiencing global collapse or extinction from a GCBR befall everyone.

GCBR mitigation also has the second requisite characteristic of publicness, in that it produces non-rivalrous benefits. One person or a whole state of people benefiting from reduced risk of artificial-pathogen pandemics or other GCBRs does not prevent others from receiving the same benefit, and nor does their use deplete the amount of risk mitigation left for others to enjoy. Note, however, that this argument is limited in specific cases where the resources required for protection against the GCBR are limited, such as where states stockpile scarce antibiotics or personal protective equipment.

As far as globalness goes, too, GCBR mitigation appears to have the requisite characteristics. Its spillover range is both relatively global and temporally extended. By definition, a *global* catastrophic biological risk threatens harm to the globe, meaning that the benefits of mitigating it reach beyond a single region. (Although, similarly to the limitations above, those benefits may not be equal across regions as in the case of the regional development and sharing of a vaccine.) What’s more, reduced risk of extinction or societal collapse from GCBRs benefits not only those who currently are at less risk of suffering, but also all of those people who might have suffered in a collapsed future society or might never have been born due to human extinction and would otherwise have had lives worth living.^1^

Regarding aggregator technologies, which one applies depends on the specific measures used for mitigation. GCBR mitigation depends on population surveillance and disaster response measures, each of which have different aggregator technologies. For instance, surveillance of DNA sequence mail-orders might be thought of as a “weakest-link” aggregator, wherein contributions to the production of the GPG are only useful insofar as every state contributes. It is not enough for all states bar one to implement effective surveillance of DNA sequence mail-orders in their countries, because all it takes is for one country to fail to implement effective surveillance for a biological warfare agent to be released there and spread in the population and on to other countries. This also indicates that many actors may be needed to produce the GPG, characterising GCBR mitigation as having the problem of jointness, another potential characteristic of public goods. Alternatively, contrast surveillance with the development of new antibiotics. In this case, it might be better to pool wealth and research resources in the country that has the best chance of successfully developing new antibiotics. A GPG of this form has a “best-shot” aggregator.

GCBR mitigation is relatively public and global. It fits the definition of a GPG. What’s more, it is similar to other established GPGs. According to Wolfgang Buchholz and Todd Sandler, “Quintessential GPGs include identifying virulent pathogens, […] eradicating infectious diseases, developing disease treatment regimes, [… and] reducing transnational terrorism” (2021, 489). GCBRs of many kinds may share essential characteristics with these quintessential GPGs. For instance, the mitigation of GCBRs seems to potentially share aggregator technologies with surveillance and vaccine/treatment development, and it shares publicness and global spillover with security from terrorism. By analogy, then, it seems GCBR mitigation also shares the label of GPG (and the related production problems) with these other cases.

Having established that GCBR mitigation constitutes a GPG, and thus that contributions to mitigation efforts constitute actions toward the production of a GPG, I now explore how this framing highlights three categories of moral reasons for individual states to contribute to mitigation, each of which I outline and evaluate in turn.

## Three Categories of Moral Reasons for Contributing to GCBR Mitigation

### Moral Nationalism

The first category of moral reason I will consider is moral nationalism. According to Locke and Hobbes, the state has particular obligations toward its citizens, by virtue of fulfilling which it is conferred legitimacy (Hoff 2015). The state brings together a political community, who give up some of their freedom in order to avoid returning to a lawless state of nature. (A Hobbesian reader may even think that the urgency of some GPG provision justifies the imposition of a world government, to bring states themselves out of a lawless state of nature (Hobbes 1651)). The purpose of the state is to provide the institutions that keep law and order, and to support citizens’ well-being. It is not common in this tradition to view states as having significant obligations toward people outside their borders. Indeed, even a welfare liberal like John Rawls limits himself  to considering justice within a state, and he explicitly rejects the possibility of the veil of ignorance extending to the global level, such that those under the veil might be motivated to ensure those elsewhere receive a minimum level of primary goods (1971). (Although he does consider justice at the international level in a limited way in later work (1999).

If the state has particular obligations to its own citizens (and, on a strongly moral nationalist view, perhaps only negligible obligations toward others) then the primary (or only) situation in which a state should contribute to the production of a GPG is when it concerns a globally shared threat or benefit whose production value for the citizens of that state alone will outweigh the costs of contribution for the state. As becomes clear through this wording, moral nationalism is essentially a moralised version of the economic argument for individual state contributions to GPGs, which, as illustrated in the section above, usually fails to motivate contributions because of the prisoner’s dilemma. One might suspect that if a state were truly a rational actor, then, at least in extreme cases like facing the threat of GCBRs, it would more often be in a state’s citizens’ interests that the state should contribute toward mitigation. However, if this is the case, it is not recognised by states, whose governments may underestimate the probability of GCBRs occurring or may be prone to the short-term thinking encouraged by short election cycles. Still, given it may be in citizens’ interests for the state to contribute to GCBR mitigation, this category of moral reason deserves further discussion.

Neil Walker seems to view nationalism—with all its limitations as a motivator for public goods coordination—as bad but inevitable. It is the economic driver that overcomes other moral reasoning much of the time (2016). Indeed, to Walker, a state that is concerned merely with protecting its own citizens’ well-being fails to recognise something important about the meaning of interconnectedness in the context of GPG production. Yet, it seems an easy fix for the production of some other public goods, particularly “instrumental” ones:

To accept the need for instrumental public goods at the global level in areas such as climate change and disease control requires only an informed and enlightened self-interest—a sense that our own community well-being depends upon the well-being of other communities being similarly secure—and a level of mutual trust sufficient to ground the credibility of common commitments made on the basis of such enlightened self-interest. (Walker 2016, 254)

For “instrumental” public goods, Walker seems to take this as the appropriate economic motivation for state action to contribute to the GPG. However, this does not apply for “communal” GPGs, which are both collectively provided and collectively enjoyed. He says, “the globe itself (or some significant part of it) counts as the relevant unit of society at which the unit-wide distribution and shared enjoyment of a good presumptively matters to all sharers” (2016, 254) for communal GPGs like living in a tolerant and secure society, even though most states fail to see this.

Which category does GCBR mitigation fall into—instrumental or communal—and what does this mean for moral nationalism as a plausible moral reason for states to engage in mitigation efforts? It might be instrumental in some senses. (Walker considers global disease control an instrumental good, after all.) But there are other benefits of GCBR mitigation that might be considered more communal—for instance, living in “a secure society.” We might think the adjective applies well to a global community that is secure from the risk of GCBRs.

It seems, from these discussions, that moral nationalism gives states only a contingent and partial reason to contribute to mitigating GCBRs. The reason is contingent insofar as it depends on benefits to the state’s citizens alone, which is by no means guaranteed in the provision of GPGs; it is partial in that even where it does benefit citizens overall, this approach of “enlightened self-interest” applies only to the provision of some benefits associated with GCBR mitigation, not for all. Thus, moral nationalism doesn’t seem like a strong reason motivating the production and maintenance of this GPG.

### Moral Cosmopolitanism

A second category of moral reason why states should contribute to GCBR mitigation relates to moral cosmopolitanism. Moral cosmopolitanism has a long history, dating back to Classical Greek thought (Kleingeld and Brown 2019). The view holds that individual states should extend moral consideration to far-away people (those outside their borders). This involves both avoiding harming others and benefiting them where feasible (and where benefits to others are not outweighed by costs or harms to the citizens of the state, on some more qualified versions) (Ferguson and Caplan 2020). The discussion is highly relevant to the production of GPGs, which can be both a means of avoiding harming others, and of benefiting them, on a global scale. In particular, moral cosmopolitanism has been linked with the mitigation of anthropogenic risks resulting from scientific progress by David Held (2010). He notes that the threats of nuclear war, terrorism, and engineered pathogens show how interdependent the world is. He claims that

… given scientific advances, the global arena becomes both an extraordinary potential space for human development as well as for disruption and destruction by individuals, groups or states (all of whom can, in principle, learn the lessons of nuclear energy, genetics, bacteriology and computer networking). (Held 2010, 296)

This is a global order in which we all belong to “overlapping communities of fate” (Held 2010, 173). The overlapping communities of fate to which Held refers are explicitly linked to global public goods and bads. In a world where we experience these goods and bads together (and recognise that fact, which may be more contentious), there is a moral reason to address them that extends beyond the imperative to protect our compatriots.^2^ This argument, applied to GCBR mitigation as a GPG, would dictate in favour of states contributing more to mitigation in order to protect the whole community of fate, the whole world.

Peter Singer, too, takes a moral cosmopolitan stance. In his *One World: The Ethics of Globalization* (2002)*,* he builds on his duty of easy rescue, previously defended as an idea of individual beneficence toward all others (1972). He uses the concept to argue for a *state* moral obligation to consider the interests of people across the world, and act accordingly when action comes at little cost. In relation to climate discussions, Singer highlights U.S. President George Bush’s failure to take an appropriately cosmopolitan stance, with the claim the United States would do nothing to reduce industrial emissions if this would negatively affect the economy because, “first things first are the people who live in America” (2002, 2). Singer contrasts this with the response of the President of the European Commission’s response, that “if one wants to be a world leader, one must know how to look after the entire earth and not only American industry” (2002, 3).

We might expect a moral cosmopolitan response to the threat of GCBRs. After all, insofar as GCBR mitigation is a GPG, there is a global public interest in its production, and it seems that the moral cosmopolitan argument provides the link necessary to establish, from this interest, a collective obligation for states to engage in GCBR mitigation efforts. However, the production and maintenance of GPGs like GCBR mitigation, even in a globalised world where we share communities of fate, suffers from two key problems that render the moral cosmopolitanism justification for state contribution somewhat weaker as a stand-alone moral reason. In examining moral cosmopolitanism as a reason for individual action, Walker claims that “we are faced with the frustrating phenomenon of one hand clapping—with a failure to reconcile authority and morality in a satisfactory manner” (2016, 251). Without an overall authority, it is unclear exactly which states have a moral obligation to contribute and exactly how those that do should contribute individually to producing the GPG (e.g., through funding a multilateral body, ensuring the production of the good in their area, or playing a direct coordination role).

As far as a moral reason for individual states to mitigate GCBRs goes, moral cosmopolitanism does establish an argument for the overall production of GPGs by states, collectively. In that way, it seems to be a less contingent and less partial moral reason than moral nationalism. Yet, it fails to be specific enough to guide state action, particularly with regard to the level of contribution that states ought to make—and which states— toward GCBR mitigation. Rather than relying on cosmopolitanism alone, it might better be supplemented by moral reasons that provide more specificity in their requirements of particular states. This supplement forms the third category of moral reason I explore: specific interstate moral obligations.

### Interstate Moral Obligations

Whilst cosmopolitanism might provide an overarching reason for states to contribute to the GPG of GCBR mitigation, there may be stronger, more specific reasons for individual states to contribute based on their relationships with other states (or other states’ citizens). Some states may not have strong moral reasons, and their appropriate contribution to a GPG may be limited. For others, there may be strong moral reasons for contributing, or a reason to make a particularly large contribution (financially, or in terms of the implementation of burdensome measures on the state’s population), on the basis of specific interstate moral obligations. Some states will have reasons to contribute to GPGs early, and others may only have reasons that come into existence after the contributions of other states. I explore these factors below, tagging interstate obligations as “first mover” or “later” obligations depending on when they ought to motivate particular states’ contributions.

First, consider a first-mover interstate obligation: moral leadership. States that aim to assert themselves as moral leaders in their region might have either altruistic or reputational reasons to provide initial contributions to GPGs like mitigating GCBRs (Safty 2003). Whilst this motivation might be considered non-moral in the reputation-building case, the *outcome* of the action (contributing to a GPG, benefiting everyone) might be considered morally good, and this might be considered moral leadership in terms of “leading on a moral action” rather than “leading in highest moral status.”

Practically, this requires that a leader state has the capacity and resources to act. It requires that they accurately judge what the right thing to do is, and that they contribute toward that goal. In the past, there have been calls for global leadership from high-income countries (HICs) that have the resources to produce or contribute to producing GPGs such as global security. However, in many cases, such countries and international groups have fallen short. Adel Safty details one such case:

In the 20th century, the League of Nations and the United Nations were founded to provide global leadership by mobilizing universal support behind a set of universal values of social progress and human development. At the end of World War I, the League of Nations held the promise of global leadership based on universal moral values such as self-determination and collective security. But the League was controlled by imperial powers more interested in furthering their respective states’ imperial interests than in providing moral leadership. […] The League became a congress of European powers determined to defend colonialism tenaciously against the rising tide of self-determination. (Safty 2003, 88)

Despite examples of failures of leadership, it acts as an effective moral reason for contributions toward GPGs in other contexts. Buchholz and Sandler raise the case of the Montreal Protocol as evidence of action by countries aiming to demonstrate moral leadership and therefore act as first movers on an issue, where they possess more resources and a significant desire to foster regard and/or regional stability. In the case of the Protocol, rich countries

… assist poor countries in meeting the required cutbacks in their use of CFCs [chlorofluorocarbons], which deplete the stratospheric ozone layer. Even though the contributions to the fund are voluntary, rich countries can anticipate that other rich countries will contribute through a moral obligation. Such contributions are side payments that foster coalition stability. Periodic reports of the Multilateral Fund indicate which rich countries are meeting their implicit obligations. (Buchholz and Sandler 2021, 515)

Particularly in cases like this, there is an emphasis on the moral leadership states might display by being the first movers. I don’t wish to imply this automatically confers greater moral authority. However, HICs may more often display moral leadership in this context insofar as they have increased capacity to perform the research required to find out where resources should be dedicated in order to most effectively contribute to a GPG, and with a larger pool of resources to contribute.

For states that have the capacity and resources for contribution, but not the aim to display moral leadership (whether for lack of reputational gain they would secure, or lack of altruism), a second, alternative interstate obligation may come into play: fairness. The Montreal Protocol example presented by Buchholz and Sandler might be explained by this interstate moral obligation, too. It may be that the environmental damage caused by many HICs today has left them more prosperous than some of their low- and middle- income country (LMIC) neighbours that have not had the opportunity to exploit these resources. This may constitute a push factor for their signing international environmental agreements, not because of the belief it will increase their reputation in a region or because they wish to contribute from altruism, but simply because they have greater capacity to contribute, they are more prosperous and more able to provide assistance in reducing CFC emissions, and it is fair that they do.

Note that this justification differs from a related concept, reparations. Whilst it may initially appear appropriate for HICs to contribute to CFC cutbacks as a way to repay LMICs for the historical overuse of shared resources HICs have engaged in, this interstate obligation is difficult to apply to the case of GPG production. It is true that many HICs today have benefited from the environmental exploitation they have been permitted to undertake up until now (Pogge 2008). The effects of such injustices are ongoing and leave the targeted people and countries with structural barriers to health, security, and other goods, and may leave them vulnerable to worse effects of public health emergencies. These are matters of extreme international injustice that we might expect to inform a reparations-based argument for contributing to GPGs like GCBR mitigation or CFC cutbacks. Indeed, this justification has featured in cases of international assistance given for disease outbreaks in the past, including the 2014–2016 Ebola outbreak in West Africa (Hooker 2014). However, there is a limitation to this interstate obligation in the case of truly *global* public goods, as all states, including the contributing HICs, benefit from the production of a GPG. Insofar as the goal of reparations is to contribute specifically to compensate the citizens of an exploited state, at a cost to the citizens of an exploiter state, this moral goal is not achieved by state contributions to GCBRs.

Let me return, then, to the argument for individual state contributions to a GPG instead on the basis of fairness. We may think that despite contributions to a GPG not being specific enough to count as an act of reparation, there is still something just in requiring prosperous states to contribute (more) toward a GPG (than less prosperous states). This might be argued by analogy to fair taxation. Much of a state’s tax income goes toward the provision of public goods for that state’s citizens. Most states favour progressive taxation, and we believe this is fair on the basis that those who are more prosperous can afford to give more to produce goods that benefit everyone. Such individuals have a moral obligation to pay their (higher) taxes on this basis. Similarly, I argue that HICs may have a moral reason to contribute (more and earlier) to GPG provision compared to LMICs, simply on the basis that they can afford to contribute more to this good that all states’ citizens benefit from. This interstate obligation is reliant on an analogy with the same rules that bind together citizens within a state, and thus on moral cosmopolitanism. I have already defended this position, so as long as it is held as an overarching reason grounding state contributions to the GPG of GCBR mitigation, this more specific interstate obligation on the basis of fairness can provide a moral reason for states to contribute to GCBR mitigation.

I have considered a number of “first mover” interstate obligations that might motivate early contributions toward a GPG. Later action may be justified for the same or other states by another interstate obligation: reciprocity. Another case study using climate change mitigation as a GPG illustrates how this final interstate obligation might motivate individual state contributions to GPGs. Paul Collier and Anthony Venables discuss the mitigation of climate change risks (2014), claiming that there are moral reasons as well as economic ones for states to close their coal mines, especially once other states have already begun to do so. They suggest “a scheme in which coal producers agree to sequence closure, with high-income countries going first” (Collier and Venables 2014, 494). This sequential aspect is essential. As established already, when there is no one yet contributing to the GPG by closing their coal mines, more appropriate moral reasons for states to contribute to the GPG include moral leadership and fairness. Once a threshold proportion of states—say, three quarters of the potential contributors—have contributed to the GPG, then the remainder have a moral pressure to contribute, because they begin to benefit from other’s (costly) contributions. Other states have, at this point, made the sacrifice, and to be one of only a few non-complying states would be a moral failure, if the state has capacity to contribute. In these kinds of cases, the Collier and Venables explain, “once the choice is framed as a moral one, most people choose not to maximize their self-interest if doing so would be unfair” (2014, 503). It is true that some states may consider this a weak moral reason. However, calls for reciprocity have not only been discussed in the case of coal mine closures but more recently have been argued to appropriately underlie state decisions to share pathogen genomic data for the purposes of controlling diseases, including COVID-19 (Silva and Smith 2023).

The same might go for moral reasons that individual states have to contribute to GCBR mitigation. There may be some states where technological developments with inadequate regulation may have already increased the risk of GCBRs. These countries might have moral leadership or fairness-based interstate obligations to make the first contributions toward mitigating GCBRs. Other states might then have increasing interstate obligations to begin their own mitigation efforts on the basis of reciprocity.

## Conclusion

Humanity may well face GCBRs such as the release of artificial pathogens with pandemic potential within the next century. States are currently not doing much to mitigate this risk, and standard economic accounts don’t seem to provide enough additional motivation to change states’ behaviour. In this paper, I propose a new argument that might be more convincing for state policymakers and publics alike.

In this work, I have established that GCBR mitigation is a GPG. That is, mitigating GCBRs produces benefits that are non-excludable, non-rivalrous, have a global spillover range, and are associated with various aggregator technologies. It is in the collective interest, then, that GCBRs are mitigated. We might even think there is a collective responsibility for states to engage in mitigation efforts, based on moral cosmopolitanism. However, more is needed to provide moral motivation for individual states.

The GPG framing is useful as it highlights three categories of potential moral reasons for mitigation. First, states should do more to mitigate GCBRs for the benefit of their own citizenries, according to moral nationalism. This provides a weak argument for contribution, however, due to the non-excludability of GPGs and their global spillover and the fact that other states might go ahead and produce the good anyway. Second, states should do more to mitigate GCBRs because, since this is a GPG, it benefits people globally, and, insofar as moral cosmopolitanism demands beneficence regardless of nationality, states should aim to benefit people the world over by mitigating GCBRs. This moral reason requires supplementation, as it lacks some specificity in the demands on different countries within the group, such that individual state obligations are unclear. Finally, specific moral motivators for state action come in the form of interstate obligations: states should do more to mitigate GCBRs as a way of fulfilling their interstate obligations, such as obligations to show moral leadership in a global region, obligations to make reparations for past injustices to other states and their citizens, and obligations of reciprocity to make their fair share of contribution toward the production of a GPG. This final category of moral reasons provides a strong foundation for the claim that individual states should do more to contribute to GCBR mitigation. I suspect that all or nearly all states will be affected by specific interstate obligations that give them moral reason to engage in efforts to mitigate GCBRs. This is relevant both to state policymakers in designing ethically informed policy on technological regulations, pandemic preparedness efforts, surveillance, and other measures that could form part of GCBR mitigation. It is also relevant to the publics of democratic states that might wish to influence their states to do more to avoid future global catastrophe.

## Conflict of Interest

The author has no conflicts of interest to declare.

## Footnotes

1 Note that I set aside questions of impersonal benefits here, and whether this case raises the non-identity problem presented by Derek Parfit (1984). It may be that GCBR mitigation affects the identities (numerical and qualitative) of future people such that the same people, by number and by character, are not born as would have been if the GCBRs had not been mitigated. In that case, the different people or people whose existence is prevented are not benefited by the risk mitigation. There are several possible responses to the non-identity problem that recognise benefits that do not befall particular people, but rather rely on comparisons between worlds (Hare 2007; Reiman 2007).

2 Note that this might also be linked to the moral reason of solidarity as holding between states. I have opted for cosmopolitanism as the more politically oriented concept that, I believe, applies better on the state level, but for an alternative framing in terms of solidarity for a similar case (preservation of the global antimicrobial commons), see de Campos-Rudinsky, 2023.
